# Investigation of aspergillosis outbreak in young ducklings: Unraveling the role of hatcheries in *Aspergillus fumigatus* transmission

**DOI:** 10.5455/javar.2023.j732

**Published:** 2023-12-31

**Authors:** Ahmed EL-Shemy, Hoda Mekky, Mohamed Bosila, Khaled Elbayoumi, Mohamed Amer, Mohamed Elaish

**Affiliations:** 1 Department of Parasitology and Animal Diseases, Veterinary Research Institute, National Research Centre, Giza, Egypt; 2 Poultry Diseases Department, Veterinary Research Institute, National Research Centre, Giza, Egypt; 3 Department of Poultry Diseases, Faculty of Veterinary Medicine, Cairo University, Giza, Egypt

**Keywords:** *Aspergillus*, hatchery, duckling, ITS gene-PCR, sequence, histopathology

## Abstract

**Objective:**

Aspergillosis is a disease that affects several species of birds and causes substantial losses in the poultry business. The purpose of the investigation was to identify the pathogen responsible for a respiratory outbreak among juvenile ducklings.

**Materials and Methods:**

An epidemic of Aspergillosis infected a total of 800 Muscovy ducks that were being reared in El-Beheira Governorate. Tissue samples were obtained to isolate suspected fungi from diseased birds and the hatchery environment. In addition, identification and molecular characterization were performed on the obtained fungal isolates.

**Results:**

Affected birds displayed acute respiratory manifestations such as difficulty breathing, gasping for air, nasal discharge, and a mortality rate of up to 28.1%. Postmortem examination revealed bronchitis, tracheitis, congested lungs, air sacculitis, severe multifocal granulomatous pneumonia, a congested, enlarged liver, and a congested kidney with nephritis. Mycological examination revealed seven *Aspergillus* (*A.)* spp. isolates from ducklings and six from hatcheries. Isolate colonial morphology and microscopical examination were as follows: *A. fumigatus, A. niger, Syncephalastrum racemosum*, and four untypable isolates. These isolates were further identified by polymerase chain reaction (PCR), and the internal transcribed spacers (ITSs) gene was detected. Four representative isolates were submitted for sequencing and further phylogenetic analysis. The source of duckling infection might be linked to the hatchery environment due to the observed similarity of isolates from both affected birds and the hatchery, as evidenced by phylogenetic analysis.

**Conclusion:**

Our findings demonstrated the significance of appropriate hatchery control in preventing infection in young ducklings. Furthermore, the use of molecular identification techniques would be helpful for tracing the source of infection and rapid diagnosis of *Aspergillus* in the field.

## Introduction

Aspergillosis is an avian affliction that is classified as noncontagious and impacts both domesticated and wild birds. It is caused by an infection with *Aspergillus* species and the vulnerability of the avian lung-air sac system to airborne pollutants and particulate matter [1]. *Aspergillus fumigatus* (*A. fumigatus*) is the most pathogenic fungus affecting poultry [2], and *Aspergillus niger (A. niger)* can also affect poultry adversely [3,^4^]*.* Infections could occur when birds are exposed to a massive number of spores, food contamination, inadequate ventilation, and bad sanitation, all of which contribute to fungal development [1]. Stressed and immune-suppressed birds are highly susceptible to disease development [2,^5^]*.*

*Aspergillus* species may infect embryos by penetrating eggshells. The infected chicks may perish or emerge with a severely developed lesion. Cracking up contaminated eggs generates spores that contaminate hatchery equipment [2]. Both *A. fumigatus and A. niger* were isolated from dead-in-shell eggs with prevalences of 28.0% and 18.6%, respectively, out of a 66.9% rate from 79 fungal isolates [6]. Exposure to environmental sources, including *in ovo,* is also implicated in infection [4]. Mortality rates during spontaneous epidemics varied between 4.5% and 90%, contingent upon the age and immune status of the avian species impacted [2]. A study reported that molds were isolated from chickens and the environment at rates of 12.8% and 25.5%, respectively, and *Aspergillus* species were the most prevalent fungi isolated [7]. In addition, several Egyptian studies have focused on aspergillosis in both humans and poultry. Results showed that the infection is widespread among those who work with infected flocks and at chicken farms and hatcheries [1,^8^].

Aspergillosis has been documented in both domestic and feral avian species. Ovo infection of the developing embryo can occur through the inhalation of conidia or spores originating from contaminated sources such as feces, sediment, and feed. In addition, contamination of eggs can lead to this infection in developing embryos. After invading the lower respiratory system (especially *A. fumigatus*) and causing symptoms including dyspnea, gasping, cyanosis, and hyperemia, infectious spores enter the respiratory tissue and proliferate, forming mycelia that cause granulomas to form. Following that, they spread via blood to different tissues [1,^9^]. In comparison to other fowl, *A. fumigatus* is the most common and noncontagious fungus that causes aspergillosis, or respiratory disease, in ducks [10]. Furthermore, ducks, particularly ducklings, are 200 times more sensitive to disease than chickens [11].

A devastating outbreak linked to pollution in the hatchery caused a 15% death rate in the first two weeks, and those who survived suffered from a delayed growth rate [12]. Acute aspergillosis in 5–10-day-old ducklings could lead to severe respiratory signs, lung lesions, and high mortality [13]. Reported mortalities in eight-day-old Pekin duckling flocks were 52%–60% from acute *Aspergillus* infection [14]. Furthermore, backyard ducks between the ages of 4 weeks and 1 year exhibit respiratory symptoms and have a death rate of up to 14% [15]*.* In addition, *A. fumigatus* caused approximately 5% mortality in 3-week-old Broiler Muscovy ducklings with a history of decreased appetite, dyspnea, and cyanotic bills [11].

Gross lesions in affected chickens showed consolidated lung parenchyma and necrotic areas on the cut surfaces of the lungs with extensive whitish discoloration and granulomatous pneumonia [2,^13^,^15^,^16^]. Air sacs showed opacity due to the localized granulomas that might coalesce [2]. Ducks infected with *A. fumigatus* showed granulomatous pneumonia due to caseous nodules in the lungs, while the liver showed granulomatous lesions due to macrophage infiltration from various pathogen infections [9,^11^].

Up until now, standard diagnostic methods have been the rule because they are easy to use and available. For example, getting a good *Aspergillus* culture from clinical tissue. Confirmatory molecular methods, such as gene sequencing using polymerase chain reaction (PCR), are used in conjunction with these traditional procedures. Given the growing number of novel species and the evolution of fungal taxonomy and nomenclature [17], molecular methods were previously used to identify different types of bacteria and fungi [18]. Phylogenetic analysis has distinct advantages for taxonomy, including the ability to group closely related isolates together despite morphological differences, which can be used to predict pathogenicity and susceptibility to antifungal drugs [19]. Internal transcribed spacers (ITSs) are excised and degraded from the ribosomal transcript during maturation. Their sequences vary more than the ribosomal sequence, making them attractive for phylogenetic analysis and/or species and strain classification [20]. The ITS region was used for the examination of clinical samples to identify the prevalence of *Aspergillus* species in poultry and poultry rearing areas and their impact on human health [1]. ITS nucleotide sequences on isolates with morphologically comparable sequences can be divided phylogenetically into different clades. These isolates were distinguished by their unique ITS nucleotide sequences, indicating the usefulness of ITS and phylogenetic analysis for the discrimination of different fungi species [21].

In this study, an outbreak of respiratory aspergillosis in ducklings was investigated, along with the isolation of fungi from ducks and hatcheries. Phylogenetic typing based on ITS sequencing was applied for accurate identification of the obtained fungal isolates and tracing back the source of the duckling infection.

## Materials and Methods

### Ethical approval

All procedures, including the use of animals in the study, were authorized by the National Research Centre Medical Research Ethics Committee, and the study was carried out in accordance with their protocols (protocol number: 20384).

### Duck flocks

An investigation was conducted on an epidemic of aspergillosis in 800 mixed-sex Muscovy ducks (*Cairina moschata*), about 10 days old, that were being raised in El-Beheira Governorate, Egypt. Swab samples were obtained from the trachea, lungs, mouth, and cloaca of diseased and dead ducklings that were 10 days old. These specimens were then cultured on Sabouraud dextrose agar medium. For histological examination, lung lesions were removed and placed in a 10% neutral buffered formalin solution.

### Histopathological examination

Lung and liver samples were collected and fixed in 10% neutral buffered formalin for the preparation of 4–6 μm-thick paraffin tissue sections. Hematoxylin and eosin were used to stain these tissue sections [22].

### Hatchery sample

Swabs (Copan ESwab^®^ transport system, Copan Diagnostics Inc., Murrieta, CA) were collected from twenty sitters and five hatcheries. The collected material was transported to the lab on swabs in a liquid Sabouraud medium with gentamycin and chloramphenicol (0.1%) added [23]. Mycological analysis was performed on the samples [24].

### Mycological examination

For the isolation and morphological identification of the obtained isolates, Sabouraud Dextrose Agar (Oxoid) was employed. Antibiotics were added to Sabouraud and glucose-potato agars, which were then inoculated with the isolates. The cultures were incubated at temperatures of 25°C and 37°C for 48 h up to 7 days to establish preliminary cultures. Distinguishing between various fungal species was achieved by assessing the conidial head and colony characteristics [25]. In addition, previously established methods for taxonomic identification were utilized [26].

### Molecular identification assays

Molecular identification techniques were applied to four representative isolates extracted from the identified samples. Briefly, DNA was extracted from samples in accordance with the manufacturer’s instructions using the QIAamp DNeasy Plant Mini reagent (Qiagen, Germany, GmbH). Nucleic acid was eluted with 50 µl of the elution buffer provided in the kit. The primer sequences used for amplification of the ITS gene were designed according to another study [27] (Table 1). PCR was performed in a 25 µl reaction containing 12.5 µl of Emerald Amp Max PCR Master Mix (Takara, Japan), 1 µl of each primer at a concentration of 20 pmol, 4.5 µl of water, and 6 µl of DNA template. The following PCR cycling conditions were used (Applied Biosystems 2720 thermal cycler): initial denaturation: 5 min at 94°C; denaturation; 30 sec at 94°C; annealing: 30 sec at 56°C, extension: 45 sec at 72°C for 35 cycles, followed by a final 10 min extension at 72°C.

**Table 1.** Primers sequences, target gene, and amplification size.

Target gene	Primers sequences	Amplified segment (bp)
ITS	ITS1: TCCGTAGGTGAACCTGCGG	~ 600
	ITS4: TCC TCC GCT TAT TGA TAT GC	

A 1.5% agarose gel (AppliChem, Germany, GmbH) was utilized to separate PCR products at room temperature in 1x TBE buffer, employing 5 V/cm gradients. The samples were examined utilizing the gene ruler 100-bp DNA ladder (Fermentas, Thermo Fisher, Germany) to evaluate the sizes of the fragments. The positive control used was *A. flavus* (ATCC^®^ 9643TM), whereas the negative control was molecular-grade water. The QIAquick PCR Product Extraction Kit was used to purify PCR products (Qiagen, Valencia, CA, USA). The sequence reaction was conducted utilizing the BigDye Terminator V3.1 Cycle Sequencing Kit (PerkinElmer, Foster City, CA, USA), and the product was purified with Centri-SepTM spin columns. An Applied Biosystems 3130 genetic analyzer (Hitachi, Japan) was used to obtain DNA sequences. A BLAST^®^ (Basic Local Alignment Search Tool) analysis [28] was conducted to establish sequence identity for GenBank accessions of the ITS gene amino acid sequence from the isolated fungus strains compared to the published strains (Fig. 3). A phylogenetic tree (Fig. 4) was generated using the CLUSTAL W multiple sequence alignment tool and the MegAlign module of Laser Gene DNASTAR version 12.1. With known sequences of the ITS gene from fungi (Table 3), phylogenetic analyses using maximum likelihood, neighbor-joining, and maximum parsimony were performed in MEGA 11 using maximum likelihood, neighbor-joining, and maximum parsimony (Table 3) [29].

**Table 2.** Published Fungi sequences of ITS gene used for multiple alignment analysis.

Number	GenBank Accession No	Isolate name
1	KX064986	*A. fumigatus* Zbf-R10
2	KP689196	*A. fumigatus* FR18
3	JF815072	*A. fumigatus* LF8
4	JN850983	*A. fumigatus* SCSGAF0014
5	JF815070	*A. fumigatus* LF6
6	JF815069	*A. fumigatus* LF5
7	JN851056	*A. fumigatus* SCSGAF0187
8	MW405811	*A. fumigatus* Shemy-AS-2
9	MW405809	*A. fumigatus* Shemy-AS-1
10	JQ316523	*A. niger* A-3207
11	KJ881376	*A. niger* MSR3
12	JQ316522	*A. niger* A-3204
13	AF455522	*A. niger* wb209
14	AF138904	*A. niger*
15	AJ280006	*A. niger* CBS 120.49
17	MW407985	*A. niger* Shemy-AS-3
17	KX898361	*A. flavus* RG01
18	MN856426	*A. flavus* WZ-309
19	MH051904	*A. cristatus* EC1
20	MG659639	*A. cristatus* ND45
21	HM999972	*S. racemosum* CBS 199.81
22	HM999973	*S. racemosum* CBS 421.63
23	MK621186	*S. racemosum* AH1
24	LC097194	*S. racemosum* M0945
25	HM999978	S. racemosum CBS 213.78
26	KF225036	*S. racemosum* UBOCC-A-101374
27	MW407961	*S. racemosum*Shemy-Syn

## Results

**Figure 1.**
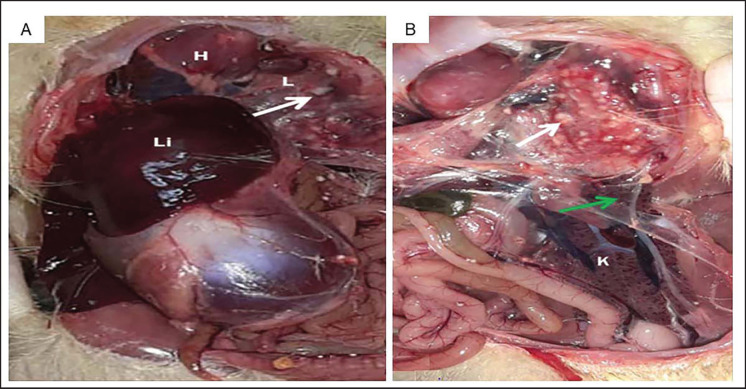
Postmortem lesion in dead 10 days Muscovy Duckling showing. (A) H: congested heart muscles, L: congested lung with whitish nodules (head of white arrow), Li: congested enlarged liver. (B) Multiple white foci (head of white arrow), Air sacculitis (head of green arrow), K: congested kidney with severe nephritis.

Muscovy ducks displayed respiratory symptoms such as gasping, dyspnea, and nasal discharge, along with a death rate of 28.1%. At postmortem examination, congested lungs, bronchitis, tracheitis, air sacculitis, severe multifocal granulomatous pneumonia, a congested enlarged liver, and a congested kidney with nephritis were detected (Fig. 1).

**Figure 2.**
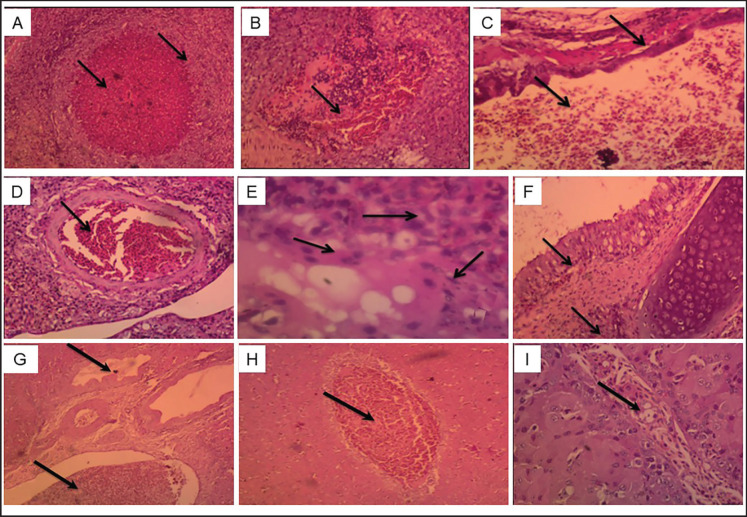
Lung and Liver sections of infected ducklings with *Aspergillus* spp. stained with H&E showing. (A) lung characteristic granuloma of aspergillosis, exhibiting central necrosis (arrow) surrounded with fibrous lining (arrow) causing atelectasis (x 100). (B) lung congestion and inflammatory cell infiltration (arrow) causing atelectasis (x 200). (C) Air sacculitis characterized by congestion and inflammatory cell infiltration (arrow) (x 200). (D) Severe congestion of the pulmonary artery (arrow) (x 200). (E) Granuloma showing septal hyphae and spores (arrows), morphologically compatible with *Aspergillus* spp., located in the necrosis areas in the central region of granulomatous lesions (arrow) (x 400). (F) Inflammation of secondary bronchi is characterized by congestion of the submucosa (arrows) and inflammatory cell infiltration (arrows) (x 200). (G) Hypertrophy of the bile duct mucosa forming finger-like projection in the lumen (arrow) and congestion of the portal vein (arrow) H&E x 200. (H) Severe congestion of the central vein (arrow) ( x 200). (I) Severe congestion of the sinusoids (arrow) ( x 300).

Histopathological examination of H&E-stained sections of lung and liver tissue samples collected from naturally infected ducklings with signs and lesions in the lungs showed a characteristic granuloma of aspergillosis, exhibiting central necrosis surrounded by fibrous lining causing atelectasis (Fig. 2A), congestion, and inflammatory cell infiltration causing atelectasis (Fig. 2B). Air sacculitis can be identified by the presence of congestion and the infiltration of inflammatory cells (Fig. 2C). Severe congestion of the pulmonary artery was recorded (Fig. 2D). Granulomatous lesion with septal hyphae and spores that look like they belong to the genus *Aspergillus*, found in the middle of necrotic granulomatous lesions (Fig. 2E). Inflammation of the secondary bronchi, characterized by congestion of the submucosa and inflammatory cell infiltration, was also seen (Fig. 2F). Liver sections from infected ducks showed hypertrophy of the bile duct mucosa, generating a finger-like projection in the lumen, as well as portal vein congestion (Fig. 2G), severe central vein congestion (Fig. 2H), and severe sinusoidal congestion (Fig. 2I).

**Figure 3.**
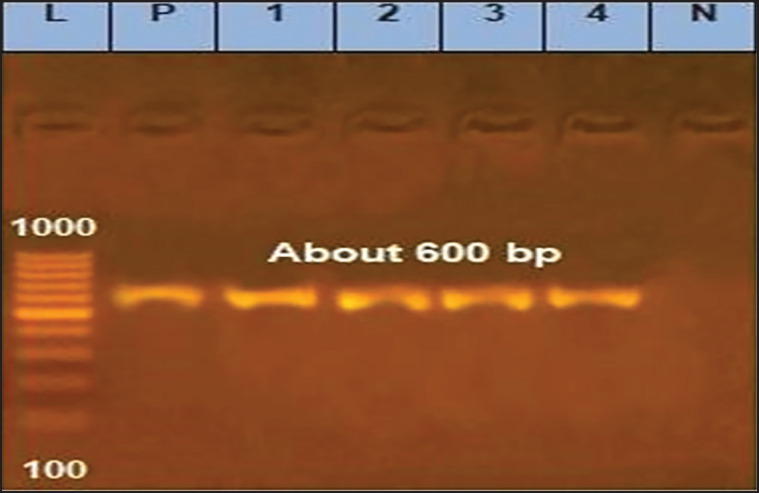
Amplified ITS gene of isolated fungi. Lane *L* = 100 bp marker; Lane P = Positive control. Lanes 1–4 = Examined samples; Lane N = Negative control.

The results of the isolation revealed seven isolates from living and dead ducklings and six from hatcheries. *A. fumigatus* (three from birds and two from the hatchery), *A. niger* (one from ducks and two from the hatchery), and *Syncephalastrum racemosum* (from the hatchery) were the five isolates that were found when the colonies were looked at under a microscope. The rest of the four isolates were untyped. PCR was utilized to further identify these isolates, and the ITS gene was detected (Fig. 3). The samples showed a 600-bp amplification of the ITS gene, which was similar to the *A. flavus* positive control used in the reaction.

**Figure 4.**
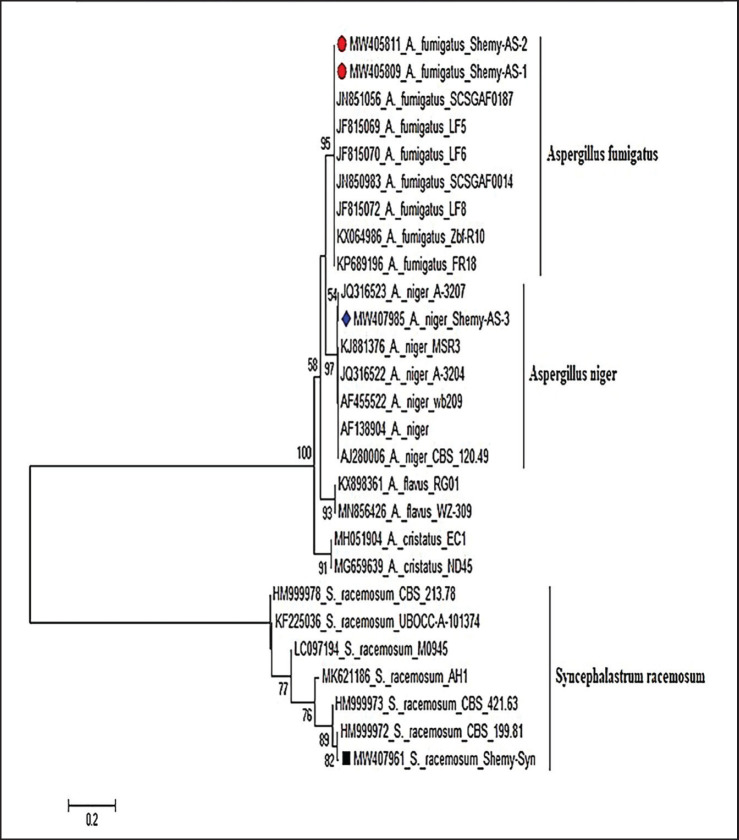
Phylogenetic tree of fungi based on the nucleotide sequence of ITS gene. Branched distances correspond to sequence divergence.

Four representative isolates (2 from ducks and two from a duck hatchery) were selected for further molecular characterization. Isolates no. 1 and 2 *A. fumigatus* (Shemy-AS-1 and Shemy-AS-2), isolate no. 3 *A. niger* (named Shemy-AS-3), and isolate no. 4. *Syncephalastrum racemosum* (named Shemy-Syn) was sequenced and submitted to NCBI. The generated phylogenetic tree, in addition to the nucleotide identity table and deduced amino acid sequence, showed homology with different percentages between our isolates in comparison to other selected twenty-three published strains in GenBank (Table 3, Fig. 4). Our *A. fumigatus* isolates were closely related to *A. fumigatus* strain SCSGAF0014, isolate LF6. Furthermore, *A. niger* strain A-3207 was closely related to our *A. nige*r isolate, while *S. racemosum* strain CBS 199.81 was identical to our Syncephalastrum isolate. Tracing back the source of duckling infection indicated that samples isolated from both hatcheries and infected ducklings were identical for *A. fumigatus, A. niger,* and *Syncephalastrum*. These results indicated that the source of the duckling infection might be linked to the contaminated hatchery environment, leading to a severe respiratory outbreak.

**Table 3.**
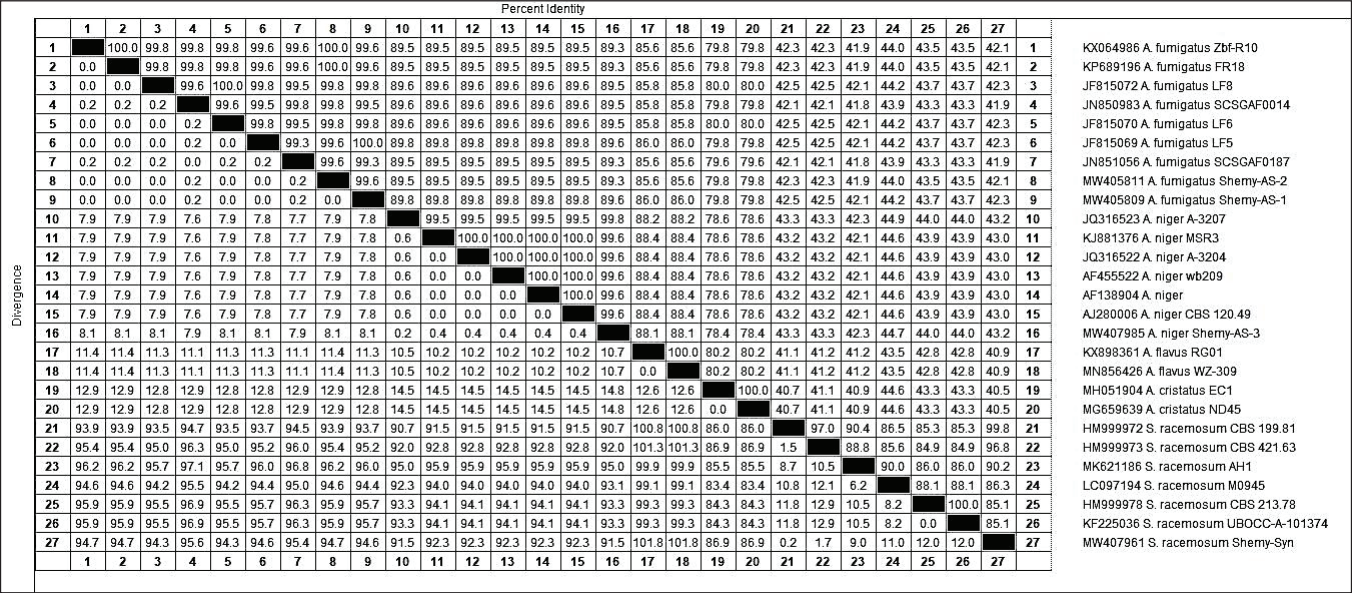
Percentage of nucleotide identities for the ITS genes of four Fungi strains named MW405809, MW405811, MW407985, and MW407961 as compared with twenty-three sequences published in GenBank.

## Discussion

The presence of severe respiratory signs such as dyspnea, gasping, hyperpnea, or bird’s depletion can serve as indications of aspergillosis, a condition primarily associated with these symptoms. However, due to the nonspecific nature of its clinical manifestations, aspergillosis can be difficult to distinguish from other diseases [30]. *Aspergillus* infections can affect birds of all species and ages, with the respiratory system being the primary site of infection [31,^32^]. *Aspergillus* infections in birds are respiratory [33,^34^]; nevertheless, additional images were also captured in the afflicted birds’ skin and neural systems [3,^35^].

A presumptive diagnosis of aspergillosis or other mycoses is based primarily on post-mortem findings of white granulomatous nodules or cheesy plaques (white caseous nodules) in the lungs, air sacs, or other visceral organs of affected birds, in addition to morphological characterization through direct examination or culture of the causative fungi. Up until now, this approach has been indispensable to classifying the isolates depending on groups that assist in identification using other methods [2,^11^,^20^,^36^].

In the current study, respiratory signs were observed in Muscovy duck flocks aged 10 days, with mortality up to 28.1%. The most recorded mortality was in young and severely affected chicks [5,^20^,^37^]. Aspergillosis causes mortalities ranging from 4.5 up to 90% in the affected avian species [3,^11^,^38^]. Macroscopically, the air sacs, lungs, liver, and kidney were affected. Mortality was 5% in natural outbreaks in ducks [11]. Lesions were observed as severe multifocal granulomatous pneumonia, congested lungs, bronchitis, tracheitis, and air sacculitis, in addition to a congested, enlarged liver and a congested kidney with nephritis [20,^39^,^40^]. Similar findings were found in commercial duck flocks as well as infected chickens, turkeys, and ducks [9,^11^,^41^].

Histopathological examination was performed on both the lungs and liver using H&E staining. Lung tissue showed characteristic granulomas of Aspergillosis, exhibiting central necrosis surrounded by fibrous lining, causing atelectasis and dyspnea in the ducks (Fig. 2A). A granulomatous lesion with septal hyphae and spores morphologically compatible with *Aspergillus* spp. was seen (Fig. 2E). Air sacculitis is characterized by congestion and inflammatory cell infiltration (Fig. 2C). On the other hand, the liver tissue showed hypertrophy of the bile duct mucosa, forming a finger-like projection in the lumen, congestion of the portal vein, severe congestion of the central vein, and sinusoids (Fig. 2G). Our findings were in concordance with the results previously reported in commercial turkeys, ducks, and chickens, respectively [5,^9^,^20^,^40^,^42^]. According to Sultana et al. [42], microscopic examination revealed several characteristic features of *A. fumigatus* infection. The observed pathological findings encompassed pulmonary and perialveolar blood vessel congestion, perivascular edema in the lungs, disseminated granulomatous foci in the lungs and air sacs, as well as infiltration of heterophils, lymphocytes, and macrophages. Furthermore, the liver tissue granuloma exhibited fatty changes, cloudy swelling, necrosis, and infiltration of red blood cells, indicating macrophage infiltration due to *A. fumigatus* infection. These findings align with the results obtained in the present study [9].

The infection with *Aspergillus* in poultry can happen if litter, the environment, or hatcheries are polluted [43]. Both warm and humid temperatures in hatcheries offer typical circumstances for the existence of *Aspergillus* [2]. In addition, unsuitable collection and storage of eggs may cause eggshell pollution. Therefore, fertile eggs and embryos may be polluted before or during incubation, and *Aspergillus* sp. is found in unhatched eggs and hatchery trash [44]. Usually, death of the infected embryos occurs between the 15th and 18th incubation days, which may lead to decreased hatchability of up to 30% [2,^4^,^44^]. Potential embryo infection occurs in dust containing more than eight hundred colonies per gram. *Aspergillus* sp. colonies were often discovered in swabs picked from hatcheries and unhatched eggs [45].

Morphological characterization serves as an initial stage in the identification of fungi and is subsequently followed by molecular methods [36]. In practice, molecular techniques such as PCR offer a rapid and minimally manipulative approach to fungal detection and characterization. These methods also aid in distinguishing between closely related fungal species based on their morphological characteristics. PCR, targeting and amplifying the highly variable ITS regions, has been widely used for fungal identification at the genus or species level [1,^20^,^46^–^49^].

In the present study, the result of mycological isolation on Sabouraud agar identified thirteen purified fungal isolates; seven fungal isolates were recovered from ducklings and six from hatcheries. Colonial morphology and microscopical identification revealed five isolates: *A. fumigatus* (3 from birds and two from hatcheries), *A. niger* (1 from ducks and two from hatcheries), and *S. racemosum* from a hatchery. The rest of the four isolates were untyped. These isolates were further identified by PCR, and the ITS gene was detected (Fig. 4). Four representative isolates (2 from ducks and two from a duck hatchery) named Shemy-AS-1, Shemy-AS-2, Shemy-AS-3, and Shemy-Syn were selected for sequencing and submitted to NCBI GenBank with accession numbers MW405809, MW405811, MW407985, and MW407961, respectively. According to ITS sequence, phylogenetic analysis, nucleotide identity, and deduced amino acids (Table 2, Fig. 4) emphasized the colonial morphology and microscopical identification of our fungal isolates. Molecular analysis indicated that our isolates were identical to the *Aspergillus* strains isolated from the hatchery. Identification of the possible source of infection as a hatchery was attributable to the similarity of ITS nucleotide sequences, as both isolates shared one common ancestor and formed one main clade, which agreed with previous reports [1,^50^]. These results also emphasized the role of the hatchery in causing the respiratory outbreak in young ducklings, in accordance with the earlier results [51,^52^]. Contaminated duck hatcheries and the environment cause early infection of embryos and ducklings at hatch and farms must be tackled with different sanitation strategies [53]. The sanitation strategies are aimed at diminishing and sustaining the number of pathogens or fungi at a suitable level [54]. Therefore, proper management is essential to prevent *Aspergillus* infection in both poultry and people. This includes keeping feedstuffs and litter as dry as possible, regularly checking the humidity level, providing appropriate ventilation, and raising knowledge of preventive and protective measures.

Our results highlighted the importance of rapid molecular methods for the diagnosis and identification of *Aspergillus* spp. in the current respiratory outbreak in young ducklings. Moreover, the current study showed the importance of proper hatchery control to avoid infection in young ducklings. To avoid contamination of embryos and ducklings at hatcheries and farms, sanitation measures such as disinfection and ventilation should be used to reduce and maintain the number of pathogens, including fungi. Moreover, appropriate prevention methods should be implemented, such as egg cleaning, fumigation, cleaning and disinfecting the hatchery on a regular basis, and a monitoring plan for the presence of *Aspergillus* in the hatchery. In the current study, hatcheries’ specific factors causing contamination and infection in ducklings were not investigated. Further research is needed to identify these factors and develop effective prevention strategies.

## Conclusion

In the current study, an outbreak of respiratory aspergillosis in ducklings was investigated. Our results showed that to prevent pollution of embryos and ducklings at hatch and farm, sanitation strategies, including disinfection and ventilation, should be performed to diminish and sustain the number of pathogens, including fungi.

## List of abbreviations

Internal Transcribed Spacers (ITS), *Aspergillus fumigatus* (*A. fumigatus*), *Aspergillus niger* (*A. niger*), polymerase chain reaction (PCR), NCBI, National Center for Biotechnology Information

## References

[1] Hassab HA, Gherbawy YA, Mubarak AG. (2019). Isolation and phylogenetic analysis of *Aspergillus* Species from birds, environment, and hospitalized patients in Qena, Egypt. AJVS.

[2] Arné P, Lee MD. (2020). Fungal infections, in diseases of Poultry.

[3] Beernaert LA, Pasmans F, Van Waeyenberghe L, Haesebrouck F, Martel A. (2010). *Aspergillus* infections in birds: a review. Avian Pathol.

[4] Kalkayeva D, Maulanov A, Sobiech P, Michalski M, Kuzembekova G, Dzhangabulova A (2023). Epidemiological characteristics and financial losses due to avian aspergillosis in households in the Almaty region, Republic of Kazakhstan. Front Vet Sci.

[5] Cheng Z, Li M, Wang Y, Chai T, Ca Yi, Li N. (2020). Pathogenicity and immune responses of *Aspergillus fumigatus* infection in chickens. Front Vet Sci.

[6] Radwan IA, Salam HSH. (2015). Fungi associated with dead-in-shell embryos of chicken and Turkey layers. Egypt J Vet Sci.

[7] Radwan IA, Abed HA, Abdallah AS. (2018). Prevalence of fungal pathogens in broiler chickens and their environment. J Vet Med Res.

[8] Radwan IA, Kamel MF, Hamdy DA, Mahmoud ZA. (2019). Correlation between *Aspergillus fumigatus* isolates recovered from human and broiler chickens. J Vet Med Res.

[9] Xue W, Li Y, Zhao Q, Liang T, Wang M, Sun P (2022). Research Note: Study on the antibacterial activity of Chinese herbal medicine against *Aspergillus flavus* and *Aspergillus fumigatus* of duck origin in laying hens. Poult Sci.

[10] Mondal D, Sahoo SK. (2018). Prevalence of duck hepatitis in an experimental farm in Bhubaneswar. Indian J Anim Health.

[11] Chung ELT, Reduan MFH, Nordin ML, Abdullah FFJ, Zairi NHM, Rajdi NZIM (2020). A case of aspergillosis outbreak in a broiler duck farm in Kelantan, Malaysia. J Adv Vet Anim Res.

[12] Brown T, Jordan FT, Wood AM. (2008). Fungal diseases. Poultry Diseases.

[13] Chung ELT, Reduan MFH, Nordin ML, Abdullah FFJ, Zairi NHM, Rajdi NZIM (2020). case of aspergillosis outbreak in a broiler duck farm in Kelantan, Malaysia. J Adv Vet Anim Res.

[14] Parker D, Walker A. (2014). Acute respiratory aspergillosis in commercial ducklings. Vet Rec Case Rep.

[15] Kaboudi K, Rejeb A, Bouzouaia M, Munir MT, Sajid U. (2018). Outbreak of respiratory aspergillosis in backyard duck flock in Tunisia. International J Livest Res.

[16] Sultana S, Rashid SMH, Islam MN, Ali MZ, Islam MM, Azam M. (2015). Pathological investigation of avian Aspergillosis in commercial broiler chicken at Chittagong district. Int J Innovat Appl Stud.

[17] Schoch CL, Seifert KA, Huhndorf S, Robert V, Spouge JL, Levesque CA (2012). Fungal barcoding consortium; fungal barcoding consortium author list. nuclear ribosomal internal transcribed spacer (ITS) region as a universal dna barcode marker for fungi. Proc Nat Acad Sci USA.

[18] Franco-Duarte R, Černáková L, Kadam S, Kaushik KS, Salehi B, Bevilacqua A (2019). Advances in chemical and biological methods to identify microorganisms-from past to present. Microorganisms.

[19] de Hoog GS, Chaturvedi V, Denning DW, Dyer PS, Frisvad JC, Geiser D (2015). ISHAM working group on nomenclature of medical fungi. Name changes in medically important fungi and their implications for clinical practice. Name changes in medically important fungi and their implications for clinical practice. J Clin Microbiol.

[20] de Oca VM, Valdés SE, Segundo C, Gómez GG, Ramírez J, Cervantes RA. (2017). Aspergillosis, a natural infection in poultry: mycological and molecular characterization and determination of gliotoxin in *Aspergillus fumigatus* isolates. Avian Dis.

[21] Abed AH, Radwan IA, El-Aziz MMA, Ali A. (2021). Antifungal activity of natural essential oils against molds and yeasts associated with respiratory problems in broiler chickens. Adv Anim Vet Sci.

[22] Bancroft JD, Layton C., Suvarna SK, Layton C, Bancroft JD (2019). The hematoxylins and eosin, in bancroft’s theory and practice of histological techniques (Eighth Edition).

[23] Gandh B, Summerbell R, Mazzulli T. (2018). Evaluation of the copan ESwab transport system for viability of pathogenic fungi by use of a modification of clinical and laboratory standards Institute Document M40-A2. J Clin Microbiol.

[24] Van Thiel DH, George M, Moore CM. (2012). Fungal infections: their diagnosis and treatment in transplant recipients. Int J Hepatol.

[25] Girma G, Abebaw M, Zemene M, Mamuye Y, Getaneh G. (2016). A review on Aspergillosis in Poultry. J Vet Sci Technol.

[26] Visagie CM, Houbraken J. (2020). Updating the taxonomy of *Aspergillus* in South Africa. Stud Mycol.

[27] Tarin NMA, Wahid MH, Ibrahim F, Yasmon A, Djauzi S. (2010). Development of multiplex-PCR assay for rapid detection of *Candida* spp. Med J Indonesia.

[28] Altschul SF, Gish W, Miller W, Myers EW, Lipman DJ. (1990). Basic local alignment search tool. J Mol Biol.

[29] Tamura K, Stecher G, Kumar S. (2021). MEGA11: molecular evolutionary genetics analysis Version 11. Mol Biol Evol.

[30] Ramírez M, Castro C, Palomares JC, Torres MJ, Aller AI, Ruiz M (2009). Molecular detection and identification of *Aspergillus* spp. from clinical samples using real-time PCR. Mycoses.

[31] Hauck R, Cray C, França M. (2020). Spotlight on avian pathology: aspergillosis. Avian Pathol.

[32] Melo AM, Silva-Filho RPd, Poester VR, von Groll A, Fernandes CG, Stevens DA (2020). Aspergillosis in free-ranging aquatic birds. Med Mycol Case Rep.

[33] Munir MT, Rehman ZU, Shah MA, Umar S. (2017). Interactions of *Aspergillus fumigatus* with the respiratory system in poultry. World’s Poult Sci J.

[34] Nururrozi A, Yanuartono Y, Widyarini S, Ramandani D, Indarjulianto S. (2020). Indarjulianto Clinical and pathological features of aspergillosis due to *Aspergillus fumigatus* in broilers. Vet World.

[35] Stoute ST, Bickford AA (2009). Walker RL, Charlton BR. Mycotic pododermatitis and mycotic pneumonia in commercial turkey poults in Northern California. J Vet Diagnost Invest.

[36] Zulkifli NA, Zakaria L. (2017). Morphological and molecular diversity of *Aspergillus* from corn grain used as livestock feed. Hayati J Biosci.

[37] Arné P, Thierry S, Wang D, Deville M, Le Loc’h G, Desoutter A (2011). *Aspergillus fumigatus* in Poultry. Int J Microbiol 2011.

[38] Silva Filho RP, Xavier MO, Martins AM, Ruoppolo V, Mendoza-Sassi RA, Adornes AC (2015). Incidence density, proportionate mortality, and risk factors of aspergillosis in magellanic penguins in a rehabilitation center from brazil. J Zoo Wildlife Med.

[39] Chu KS, Kang MS, Lee JW. (2012). A case of Aspergillosis in commercial domestic ducks. Korean J Vet Service.

[40] Kaboudi K, Rejeb A, Bouzouaia M, Munir MT, Sajid U. (2018). Outbreak of respiratory aspergillosis in backyard duck flock in Tunisia.

[41] Singh SP, Borah M, Sharma DK, Joshi GD, Gogoi R. (2009). Aspergillosis in turkey poults. Indian J Vet Patholol.

[42] Sultana S, Rashid SM, Islam MN, Ali MH, Islam MM, Azam MG. (2015). Pathological investigation of avian aspergillosis in commercial broiler chicken at Chittagong district. Int J Innovat Appl Stud.

[43] Dyar PM, Fletcher OJ, Page RK. (1984). Aspergillosis in turkeys associated with use of contaminated litter. Avian Dis.

[44] Jacobsen ID, Große K, Slesiona S, Hube B, Berndt A, Brock M. (2010). Embryonated eggs as an alternative infection model to investigate *Aspergillus fumigatus* virulence. Infect Immun.

[45] Kapetanov MC, Potkonjak DV, Milanov DS, Stojanov IM, Živkov-Baloš MM, Prunić B. (2011). Investigation of dissemination of aspergillosis in poultry and possible control measures. Zbornik Matice srpske za prirodne nauke.

[46] Abd El Tawab AA, Maarouf AAA, El-Hofy FI, Ahmed KSM. (2015). Molecular characterization of some fungi isolated from broiler chicken farms. Benha Vet Med J.

[47] Faber J, Moritz N, Henninger N, Zepp F, Knuf M. (2009). Rapid detection of common pathogenic *Aspergillus* species by a novel real-time PCR approach. Mycoses.

[48] Fagbohun ED, Ayantola KJ, Toyin-Famoroti AJ. (2020). Isolation and molecular characterization of *Aspergillus fumigatus* and *Aspergillus flavus* isolated from poultry birds in Ado-Ekiti, Nigeria. Asian J Biotechnol Bioresour Technol.

[49] Ulloa-Avellán O, Calderón-Hernández A, Rubí-Chacón R, Vargas-Leitón B. (2023). *Aspergillus* spp. isolated from lungs of poultry (*Gallus gallus*) at the Mycology Laboratory, School of Veterinary Medicine, Universidad Nacional, Heredia, Costa Rica between 2008 and 2021 and associated factors. J Fungi.

[50] Mkumbe BS, Sajidan, Pangastuti A, Susilowati A. (2018). Phylogenetic analysis based on internal transcribed spacer region (ITS1-5.8S-ITS2) of *Aspergillus niger* producing phytase from Indonesia. AIP Conf Proc.

[51] Wright ML, Anderson GW, Epps NA. (1960). Hatchery Sanitation as a control measure for aspergillosis in fowl. Avian Dis.

[52] Hamet N, Seigle-Murandi F, Steiman R. (1991). Contribution to the prophylaxis of chicks aspergillosis: study of the contamination of a hatchery by *Aspergillus fumigatus*. Zent Vet B.

[53] Oliveira GD, McManus C, Salgado CB, dos Santos VM. (2022). Effects of sanitizers on microbiological control of hatching eggshells and poultry health during embryogenesis and early stages after hatching in the last decade. Animals (Basel).

[54] Abd El-Hack ME, Hurtado C, Toro DM, Alagawany M, Abdelfattah EM, Elnesr SS. (2019). Fertility and hatchability in duck eggs. World’s Poult Sci J.

